# CCDC137 Is a Prognostic Biomarker and Correlates With Immunosuppressive Tumor Microenvironment Based on Pan-Cancer Analysis

**DOI:** 10.3389/fmolb.2021.674863

**Published:** 2021-05-13

**Authors:** Lihao Guo, Boxin Li, Zhaohong Lu, Hairong Liang, Hui Yang, Yuting Chen, Shiheng Zhu, Minjuan Zeng, Yixian Wei, Tonggong Liu, Tikeng Jiang, Mei Xuan, Huanwen Tang

**Affiliations:** Dongguan Key Laboratory of Environmental Medicine, Department of Environmental and Occupational Health, School of Public Health, Guangdong Medical University, Dongguan, China

**Keywords:** CCDC137, pan-cancer, tumor associated macrophages, immunosuppression status, tumor microenvironment

## Abstract

**Background:**

The coiled-coil domain containing (CCDC) family proteins have important biological functions in various diseases. However, the coiled-coil domain containing 137 (CCDC137) was rarely studied. We aim to investigate the role of CCDC137 in pan-cancer.

**Methods:**

CCDC137 expression was evaluated in RNA sequence expression profilers of pan-cancer and normal tissues from The Cancer Genome Atlas (TCGA) and Genotype-Tissue Expression (GTEx) database. The influence of CCDC137 on the prognosis of tumor patients was analyzed using clinical survival data from TCGA. Function and pathway enrichment analysis was performed to explore the role of CCDC137 using the R package “clusterProfiler.” We further analyzed the correlation of immune cell infiltration score of TCGA samples and CCDC137 expression using TIMER2 online database.

**Results:**

CCDC137 was over-expressed and associated with worse survival status in various tumor types. CCDC137 expression was positively correlated with tumor associated macrophages (TAMs) and cancer associated fibroblasts (CAFs) in Lower Grade Glioma (LGG) and Uveal Melanoma (UVM). In addition, high CCDC137 expression was positively correlated with most immunosuppressive genes, including TGFB1, PD-L1, and IL10RB in LGG and UVM.

**Conclusions:**

Our study identified CCDC137 as an oncogene and predictor of worse survival in most tumor types. High CCDC137 may contribute to elevated infiltration of TAMs and CAFs and be associated with tumor immunosuppressive status.

## Introduction

Tumor microenvironment (TME) has been proved to be composed of complex components, and is crucial for tumor development and prognosis of patients (Cassim and Pouyssegur, 2019). The immune and stromal cells within TME have essential contributions to tumorigenesis and development of tumor (Lei et al., 2020). Tumor associated macrophages (TAMs) could secrete several cytokines and chemokines to alleviate tumor immunity and promote tumor progression. Moreover, the extensive heterogeneity of TAMs enables immune and stromal cells to adapt or alter their phenotypes to conform to the TME, playing an oncogene role in cancer progression and metastasis (Pathria et al., 2019). In addition, cancer associated fibroblasts (CAFs) in TME have abilities to secrete various growth factors, cytokines, and chemokines to promote tumor progression. TGFB1, which was mainly secreted by CAFs and TAMs, was one of the main inducements of tumor immunosuppressive microenvironment (Liao et al., 2019). Therefore, it is urgent to explore the potential molecular mechanism leading to the formation of CAFs and TAMs.

The coiled-coil domain containing (CCDC) family proteins have important biological functions. Many members of CCDC family are involved in the regulation of invasion and metastasis of malignant tumor cells. At present, it has been confirmed that the abnormal expression of CCDC34, CCDC67, and CCDC88A proteins can significantly affect the malignant progress of lung cancer, bladder cancer, thyroid cancer, and pancreatic cancer (Park et al., 2012; Gong et al., 2015; Tanouchi et al., 2016; Geng et al., 2018), which provides a theoretical basis for the application of new medicine in tumor targeted therapy.

CCDC137, a member of CCDC family proteins, was rarely studied in previous researches. In this study, we performed a pan-cancer analysis to explore the role of CCDC137 in tumor progression. In detail, we analyzed the expression level, methylation level, copy-number value, mutation status, and prognostic value of CCDC137 in TCGA pan-cancer. In addition, the associations between CCDC137 and tumor stromal cells, tumor-infiltrating immune cells, and immune related marker genes were investigated. This study revealed the potential role of CCDC137 in TME and its prognostic value in pan-cancer, which may help explore a new drug target.

## Materials and Methods

### Data Collection

The RNA expression profiles and patient survival information of pan-cancer data of TCGA database were downloaded from UCSC-XENA^1^. The RNA expression profiles of The Genotype-Tissue Expression (GTEx) were downloaded from UCSC-XENA. The methylation level and copy-number value of CCDC137 in TCGA pan-cancer were downloaded from cBioportal^2^.

### Data Analysis Tools

TIMER2^3^ database was used to draw expression difference of CCDC137 using TCGA pan-cancer data. cBioportal database was used to show the alteration frequency of CCDC137 using TCGA data. R packages “survival,” and “survminer” were employed to perform Kaplan–Meier survival analysis. For functional enrichment analysis, R package “clusterprofiler” was used to perform Gene Set Enrichment Analysis (GSEA) analysis. Ualcan^4^ database was used to evaluate protein and protein phosphorylation level of CCDC137. TISIDB database^5^ was used to analysis CCDC137 expression in different molecular subtypes of tumor samples from TCGA.

### Immune Cell Infiltration Analysis

TIMER2 database was used to analyze associations between CCDC137 and tumor stromal cells, tumor-infiltrating immune cells. The immunosuppressive gene was obtained from published paper “Pan-cancer Immunogenomic Analyses Reveal Genotype-Immunophenotype Relationships and Predictors of Response to Checkpoint Blockade” (Charoentong et al., 2017).

## Results

### Pan-Cancer CCDC137 Expression

**FIGURE 1:**
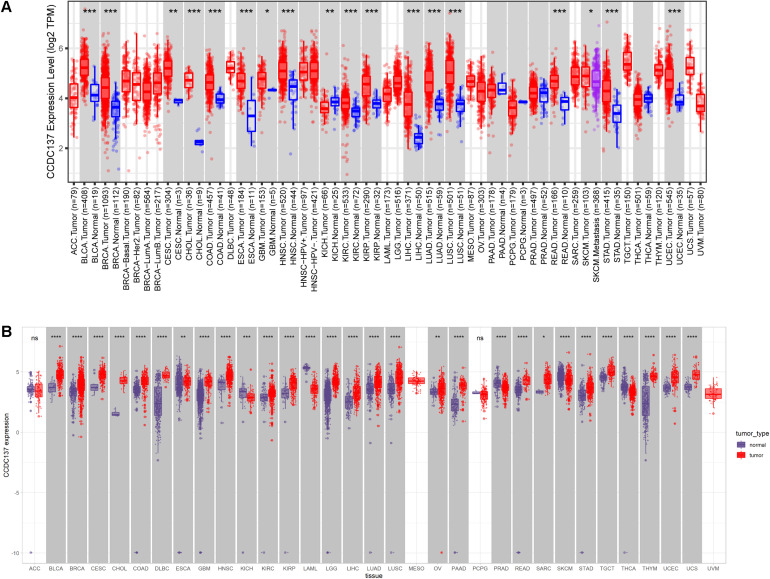
Pan-cancer CCDC137 expression analysis. **(A)** CCDC137 expression in tumor and adjacent normal tissues in pan-cancer data of TCGA cohort. **(B)** CCDC137 expression in tumor tissues from TCGA and normal tissues from TCGA and GTEx cohorts. Data shown as mean ± SD. *p < 0.05, **p < 0.01, ***p < 0.001, and ****p < 0.0001.

We first evaluated the mRNA expression of CCDC137 in pan-cancer data of TCGA using TIMER2 database. Results revealed that CCDC137 was highly expressed in 16 tumor types including Bladder Urothelial Carcinoma (BLCA), Breast invasive carcinoma (BRCA), Cervical squamous cell carcinoma and endocervical adenocarcinoma (CESC), Cholangiocarcinoma (CHOL), Colon adenocarcinoma (COAD), Esophageal carcinoma (ESCA), Glioblastoma multiforme (GBM), Head and Neck squamous cell carcinoma (HNSC), Kidney renal clear cell carcinoma (KIRC), Kidney renal papillary cell carcinoma (KIRP), Liver hepatocellular carcinoma (LIHC), Lung adenocarcinoma (LUAD), Lung squamous cell carcinoma (LUSC), Rectum adenocarcinoma (READ), Stomach adenocarcinoma (STAD), and Uterine Corpus Endometrial Carcinoma (UECE), while only low expressed in Kidney Chromophobe (KICH) (Figure 1A). As the number of normal tissues in TCGA is limited, we further analyzed the CCDC137 expression combining normal tissue data of GTEx database with TCGA data. We found that CCDC137 was over-expressed in 24 tumor types. In addition to 16 tumor types mentioned above, there are also Lymphoid Neoplasm Diffuse Large B-cell Lymphoma (DLBC), Brain Lower Grade Glioma (LGG), Ovarian serous cystadenocarcinoma (OV), Pancreatic adenocarcinoma (PAAD), Sarcoma (SARC), Testicular Germ Cell Tumor (TGCT), Thymoma (THYM), and Uterine Carcinosarcoma (UCS). While CCDC137 was low expressed in KICH, Acute Myeloid Leukemia (LAML), Prostate adenocarcinoma (PRAD), Skin Cutaneous Melanoma (SKCM), Thyroid carcinoma (THCA) (Figure 1B).

**FIGURE 2:**
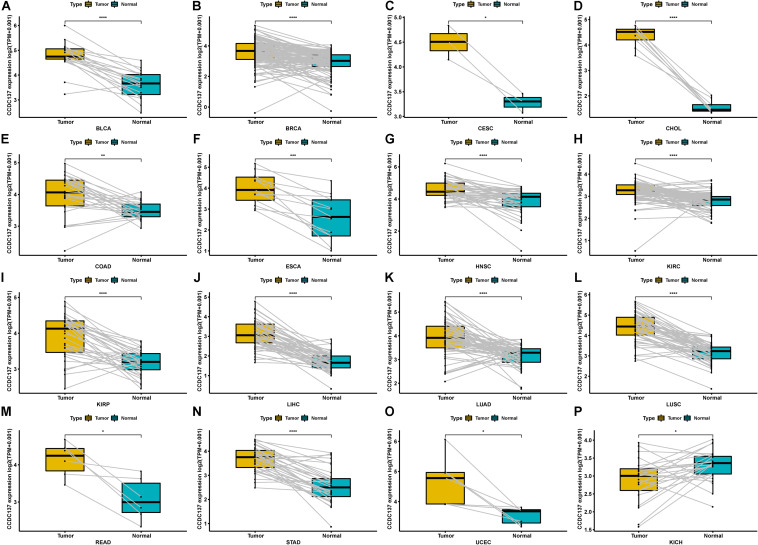
Pan-cancer CCDC137 expression in paired tumor and normal tissues. **(A–P)** CCDC137 expression in paired tumor and adjacent normal tissues in indicated tumor types in TCGA cohort. Data shown as mean ± SD. *p < 0.05, **p < 0.01, ***p < 0.001, and ****p < 0.0001.

In addition, for paired tumors and adjacent normal tissues in TCGA, CCDC137 was over-expressed in tumor tissues of BLCA, BRCA, CESC, CHOL, COAD, ESCA, HNSC, KIRC, KIRP, LIHC, LUAD, LUSC, READ, STAD, and UCEC (Figures 2A–O), while low-expressed in KICH (Figure 2P). We then analyzed the CCDC137 expression in different WHO stages and molecular subtypes. We found that CCDC137 expression was higher in relative worse tumor stages in BRCA, LUSC, KIRC, KICH, and HNSC (Supplementary Figures 1A–E). We also observed that CCDC137 expression was significantly different in different molecular subtypes of BRCA, HNSC, LGG, LUSC, OV, PCPG, PRAD, STAD, and UCEC (Supplementary Figures 1A–N).

### DNA Methylation and CNA Alterations of CCDC137 in TCGA Pan-Cancer

**FIGURE 3:**
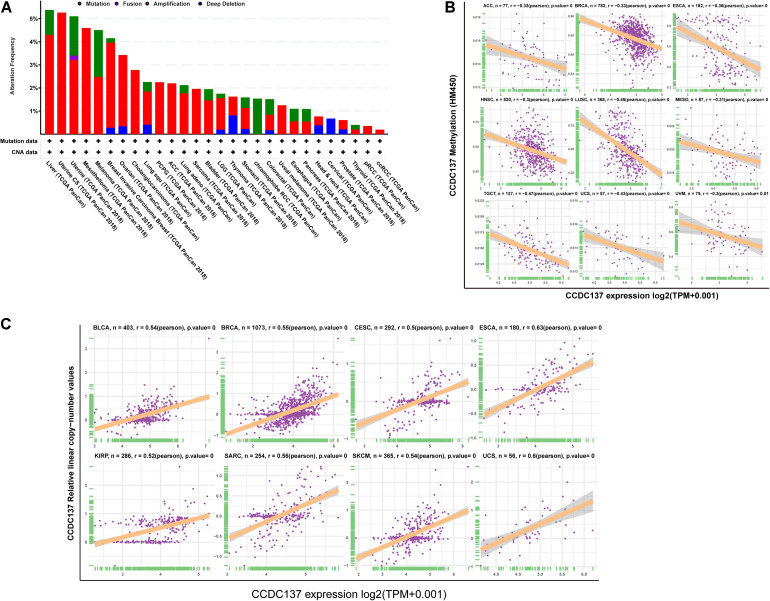
Pan-cancer analysis of CNA and DNA methylation of CCDC137. **(A)** CNA and mutation frequency of CCDC137 in TCGA pan-cancer were accessed using cBioPortal. **(B)** The correlation between DNA methylation and mRNA expression of CCDC137 in TCGA pan-cancer. **(C)** The correlation between CNA and mRNA expression of CCDC137 in TCGA pan-cancer.

To explore the reasons of the high CCDC137 expression in tumor, we evaluated the genetic and epigenetic changes of CCDC137 using TCGA data from cBioPortal. We found that patients with high CCDC137 expression were accompanied by high gene alterations in LIHC, UCEC, BRCA, OV, CHOL, LUSC, and LUAD (Figure 3A). For the association between DNA methylation level and mRNA expression of CCDC137, we found that DNA methylation level was significantly negatively correlated with CCDC137 expression in nine tumor types, including ACC, BRCA, ESCA, HNSC, LUSC, MESO, TGCT, UCS, and Uveal Melanoma (UVM) (Figure 3B). In addition, we further analyzed the association between relative linear copy number values and mRNA expression of CCDC137. The results revealed a significant positive correlation between CCDC137 expression and copy number variation (CNA) in BLCA, BRCA, CESC, ESCA, KIRP, SARC, SKCM, and UCS (Figure 3C).

### Protein and Protein Phosphorylation Alterations of CCDC137 in TCGA Pan-Cancer

**FIGURE 4:**
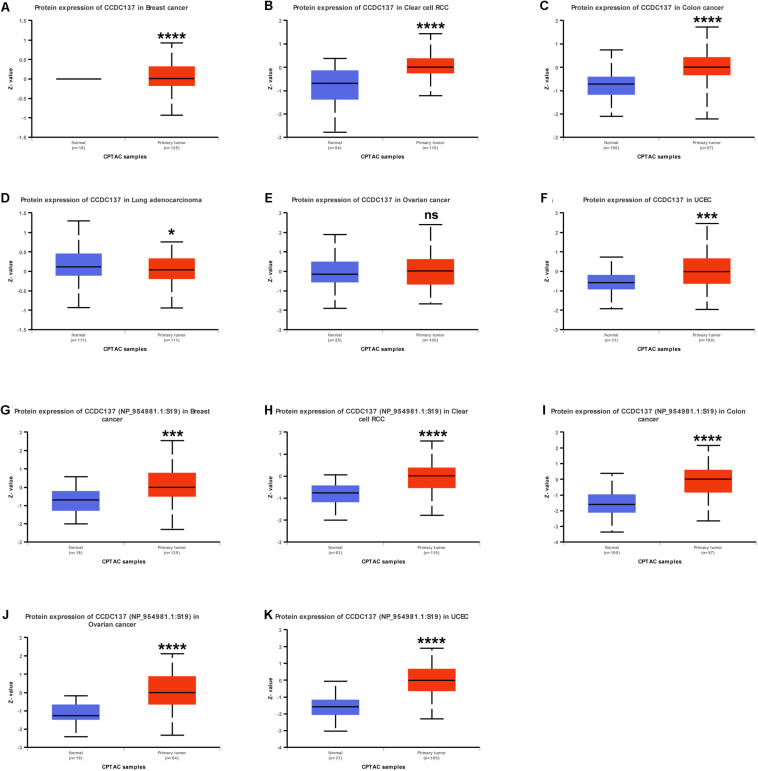
Pan-cancer protein analysis of CCDC137. **(A–F)** CCDC137 protein level in indicated tumor types from TCGA cohort. **(G–K)** CCDC137 protein phosphorylation level in indicated tumor types from TCGA cohort. Data shown as mean ± SD. *p < 0.05, **p < 0.01, ***p < 0.001, and ****p < 0.0001.

Since protein expression level is the key factor directly affecting molecular function, we further analyzed the protein and protein phosphorylation level of CCDC137 in TCGA pan-cancer using Ualcan database. The results revealed that the protein level of CCDC137 was higher in tumor tissues than that in normal tissues in BRCA, KIRC, COAD, LUAD, and UCEC, while no difference in OV (Figures 4A–F). In addition, we found that there was only one phosphorylation site of CCDC137. High phosphorylation level of CCDC137 was observed in BRCA, KIRC, COAD, OV, and UCEC (Figures 4G–K).

### Prognostic Value of CCDC137 in TCGA Pan-Cancer

**FIGURE 5:**
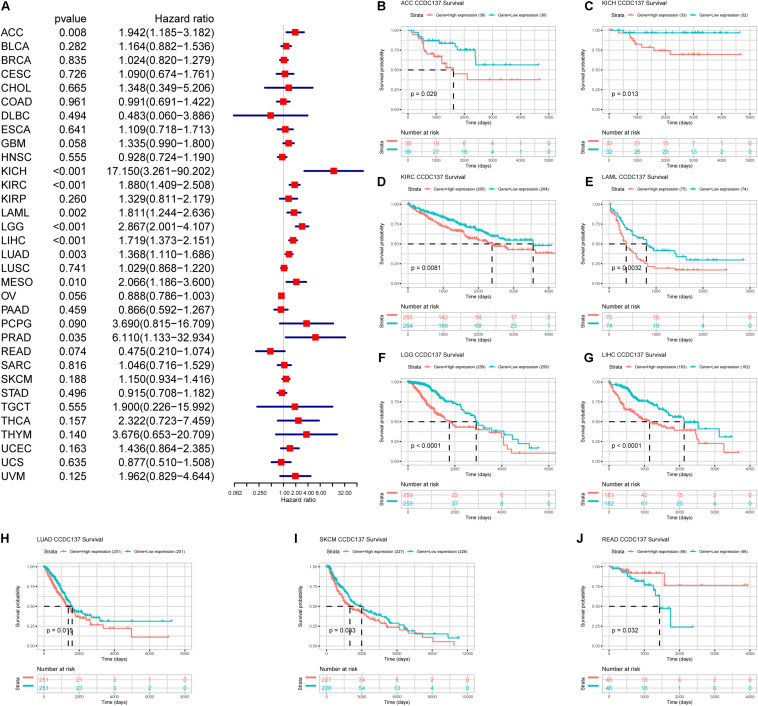
Prognosis value of CCDC137 in TCGA pan-cancer. **(A)** The forest map shows the results of Univariate Cox Regression analysis for OS. **(B–J)** Kaplan–Meier survival analysis of CCDC137 in indicated tumor types. Only tumor types with log rank *p* < 0.05 were displayed.

Next, we investigated the prognostic value of CCDC137 in TCGA pan-cancer using Univariate Cox Regression analysis and Kaplan–Meier analysis. The Univariate Cox Regression analysis revealed that high expression of CCDC137 was a risk factor of overall survival (OS) in ACC, KICH, KIRC, LAML, LGG, LIHC, LUAD, MESO, and PRAD (Figure 5A). For disease free interval (DFI), higher expression of CCDC137 was associated with poorer DFI in ACC, LGG, LIHC, and PRAD (Supplementary Figure 2A). For progression free interval (PFI), higher expression of CCDC137 was associated with reduced PFI in ACC, KICH, KIRC, LGG, LIHC, LUSC, MESO, PCPG, PRAD, and UVM, while increased PFI in OV (Supplementary Figure 2B). For disease-specific survival (DSS), higher expression of CCDC137 was associated with worse DSS in ACC, KICH, KIRC, LGG, LIHC, LUAD, MESO, PCPG, PRAD, THCA, and THYM, while better DSS in OV (Supplementary Figure 2C). The Kaplan–Meier analysis suggested that high CCDC137 expression predicted poor OS in ACC, KICH, KIRC, LAML, LGG, LIHC, LUAD, and SKCM, while longer OS time in READ (Figures 5B–J).

### Immune Cell Infiltration Analysis

**FIGURE 6:**
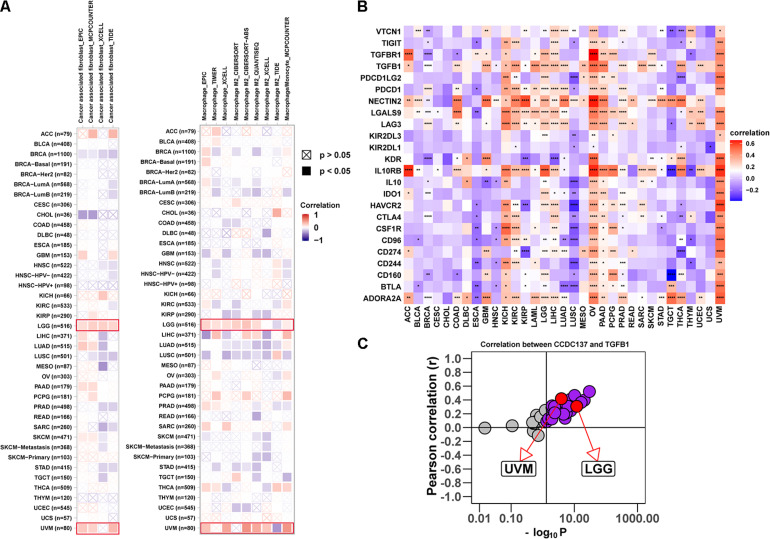
Analysis of the effect of CCDC137 on immune microenvironment. **(A)** The correlation between CCDC137 expression and CAFs (left) or TAMs (right) were shown. Red color represents positive correlation, blue color represents negative correlation, and the deeper the color, the stronger the correlation. **(B)** The heatmap shows the correlation between CCDC137 and immunosuppressive genes in TCGA pan-cancer. **(C)** Correlation coefficient and –log10 (*p* value) of CCDC137 with TGFB1 is shown. Each circle represents a different tumor type from TCGA. Red circles are marked for UVM and LGG. Gray circles mean no correlation.

Tumor associated macrophages and CAFs, as prominent components of TME, were closely related to the occurrence, development, and metastasis of tumor. Thus, we further investigated the association between CCDC137 expression and infiltration level of TAMs and CAFs using TIMER2 online database. We observed a positive correlation between the infiltration of TAMs/CAFs and CCDC137 expression in the tumors of LGG and UVM based on all or most algorithms (Figure 6A). We further selected 24 immunosuppressive marker genes based on published article and performed the correlation analysis with CCDC137 (Charoentong et al., 2017). The results revealed that 17 of 24 immunosuppressive marker genes was positively correlated with CCDC137 expression in LGG and 22 of 24 immunosuppressive marker genes was positively correlated with CCDC137 expression in UVM (Figure 6B). In these immunosuppressive marker genes, TGFB1, NECTIN2, LGALS9, LAG3, and IL10RB were significantly correlated with CCDC137 expression in most tumor types. As we have known, there was a significant correlation between TGFB1 expression and TAMs/CAFs. We observed that CCDC137 was significantly correlated with TGFB1 expression in most tumor types including UVM and LGG, which may indicate the potential mechanism of CCDC137 influencing infiltration of TAMs/CAFs (Figure 6C).

### Gene Set Enrichment Analysis of CCDC137

**FIGURE 7:**
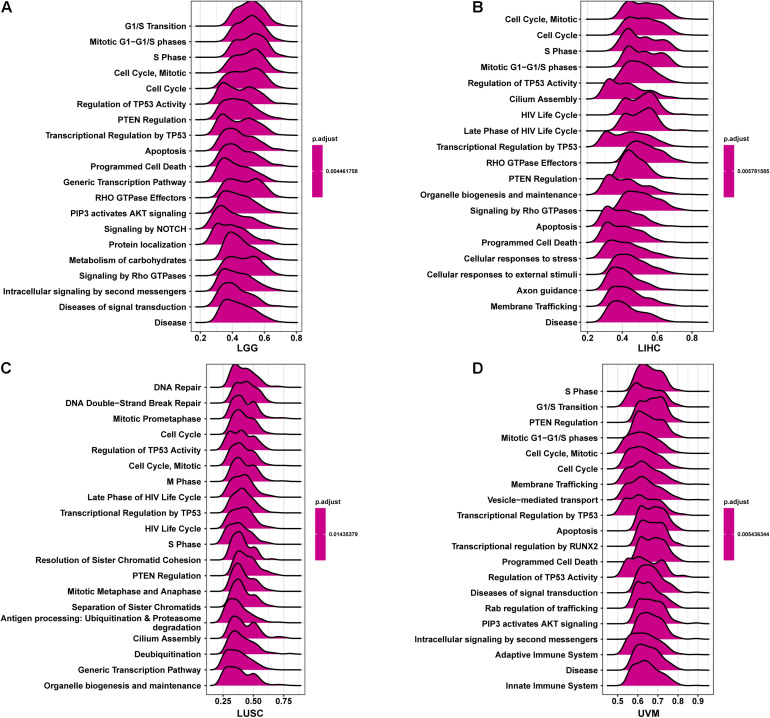
GSEA of CCDC137 in TCGA pan-cancer. **(A–D)** The top 20 GSEA results of CCDC137 in indicated tumor types from TCGA.

To better explore the pathways CCDC137 may participate in, we conducted GSEA using R package “clusterprofiler.” We observed that CCDC137 was mainly enriched in cell cycle related pathways in most tumor types. For example, CCDC137 was enriched in G1/S Transition in LGG, Cell Cycle, Mitotic in LIHC, Cell Cycle in LUSC, and S Phase in UVM (Figures 7A–D). These results indicated that CCDC137 was a major participant in tumor cell cycle process, which provided a potential direction for future research.

## Discussion

The CCDC family proteins have important biological functions in various diseases. For example, CCDC43 was proved to accelerate proliferation and metastasis process of gastric cancer (Wang et al., 2020). In addition, CCDC25 was recently observed to promote cancer metastasis (Yang et al., 2020). However, CCDC137 was rarely studied for now.

In our study, we examined the CCDC137 mRNA and protein expression levels and prognostic value in pan-cancer using TCGA and GTEx data downloaded from UCSC Xena. Based on our results, we found that CCDC137, compared to normal tissues, was over-expressed in 16 tumor types including BLCA, BRCA, CESC, CHOL, COAD, ESCA, GBM, HNSC, KIRC, KIRP, LIHC, LUAD, LUSC, READ, STAD, and UECE. High CCDC137 expression predicts poor OS in ACC, KICH, KIRC, LAML, LGG, LIHC, LUAD, MESO, and PRAD.

Tumor microenvironment, especially tumor immune and stromal microenvironment, constitute a vital element of tumor tissue. Increasing evidence has revealed their clinicopathological significance in predicting outcomes and therapeutic efficacy (Greten and Grivennikov, 2019; Vitale et al., 2019; Suzuki et al., 2021). TAMs and CAFs in TME always accelerate tumor progression (Akins et al., 2020; Liu et al., 2021; Shan et al., 2021). Our results revealed that CCDC137 have close relationships with TAMs and CAFs infiltration in most tumor types. Moreover, the positive correlations between CCDC137 expression and immunosuppressive genes, such as TGFB1, NECTIN2, LGALS9, LAG3, and IL10RB, indicate the key role of CCDC137 in regulating tumor immunology, macrophage polarization, and CAFs formation. TAMs, which are particularly abundant in a tumor mass, contribute much to the immunosuppressive microenvironment (Zeng et al., 2021). TGFB1, mainly secreted by TAMs and CAFs in TME, play an irreplaceable role in inducing immunosuppressive microenvironment. Immunosuppressive genes, such as IL10, IL10RB, LGALS9, and LAG3, were observed to be co-overexpressed with TGFB1 in tumor tissues and predicted poor survival of tumor patients, indicating a potential mechanism by which CCDC137 regulates macrophage polarization, CAFs formation and correlates with several immunosuppressive genes (Kadowaki et al., 2016; Fan et al., 2020; Suzuki et al., 2021; Wang et al., 2021). In addition, the high expression of CCDC137 indicates the immunosuppression status in LGG and UVM, providing a potential drug target for tumor therapy.

In conclusion, CCDC137 may play an important role in macrophage polarization and CAFs formation in TME. Targeting CCDC137 may become a potential treatment for cancer.

## Data Availability Statement

The datasets presented in this study can be found in online repositories. The names of the repository/repositories and accession number(s) can be found in the article/ Supplementary Material.

## Author Contributions

LG: conceptualization, methodology, software, formal analysis, writing-original draft, and visualization. BL and ZL: formal analysis, software, visualization, investigation, and validation. HL, HY, and YC: software, validation, and investigation. SZ, MZ, YW, TL, TJ, and MX: investigation and data curation. HT: conceptualization, methodology, writing-review and editing, supervision, and funding acquisition. All authors contributed to the article and approved the submitted version.

## Conflict of Interest

The authors declare that the research was conducted in the absence of any commercial or financial relationships that could be construed as a potential conflict of interest.

## Footnotes

1 https://xenabrowser.net/

2 https://www.cbioportal.org/

3 http://timer.cistrome.org/

4 http://ualcan.path.uab.edu/index.html

5 http://cis.hku.hk/TISIDB/index.php
