# Supplementary material for: CCDC137 Is a Prognostic Biomarker and Correlates With Immunosuppressive Tumor Microenvironment Based on Pan-Cancer Analysis
Source: Front Mol Biosci. 2021 May 13;8:674863. doi: 10.3389/fmolb.2021.674863 (PMC8155610; doi:10.3389/fmolb.2021.674863)
Supplement: Supplementary file 1 [file Data_Sheet_1.docx]

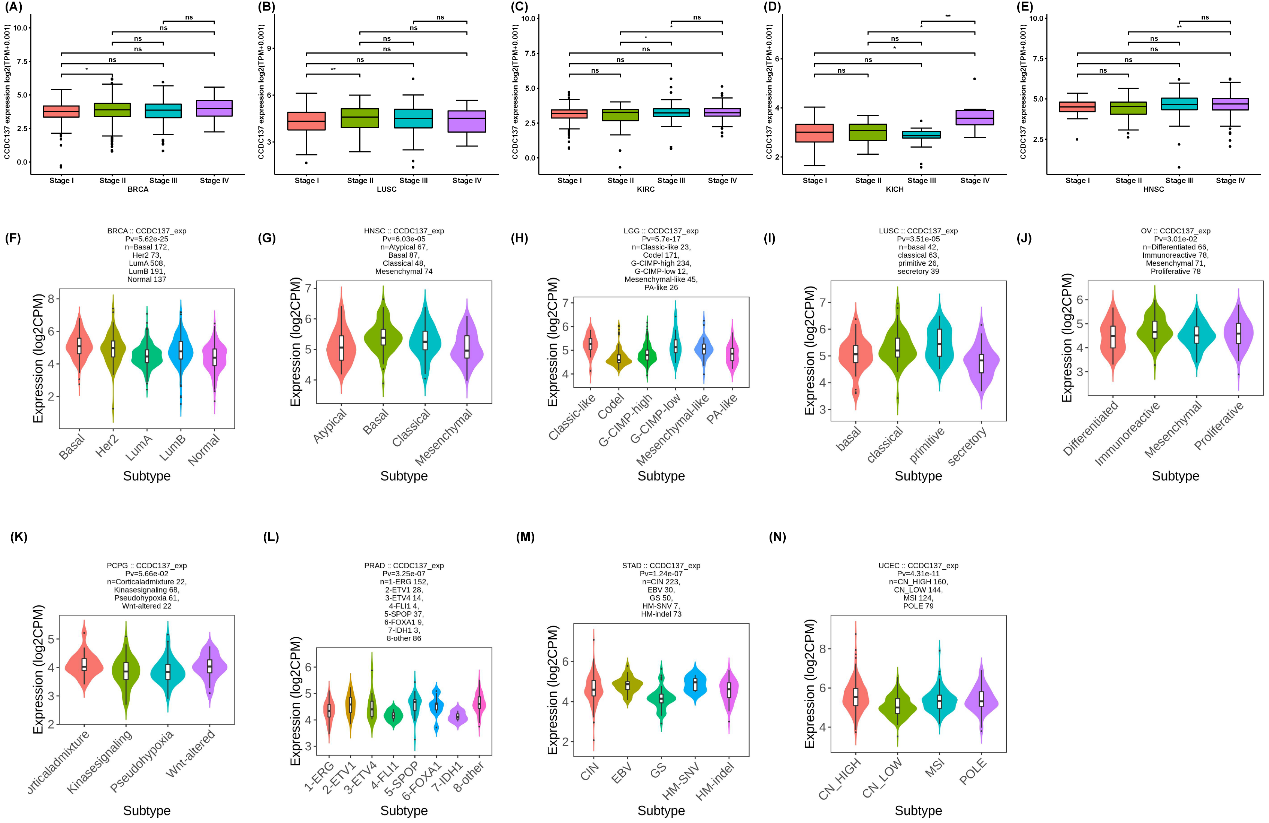


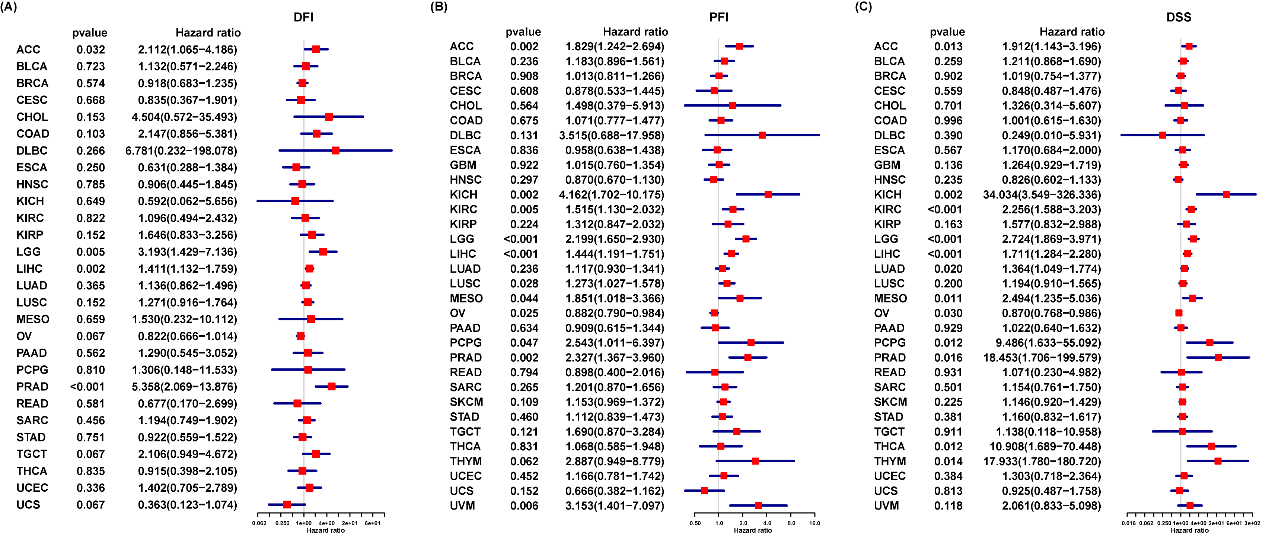


**Supplementary figure legends**

**Sup-Figure 1: CCDC137 expression.** (A-E) CCDC137 expression in indicated stages of tumor samples from TCGA. (F-N) CCDC137 expression in indicated different molecular subtypes of tumor samples from TCGA. Data shown as mean ± SD. *p < 0.05, **p < 0.01, ***p < 0.001, ****p < 0.0001.

**Sup-Figure 2: Univariate Cox Regression analysis of CCDC137.** (A) The forest map shows the results of Univariate Cox Regression analysis for DFI. (B) The forest map shows the results of Univariate Cox Regression analysis for PFI. (C) The forest map shows the results of Univariate Cox Regression analysis for DSS.
